# Can Supervised Pelvic Floor Muscle Training Through Gametherapy Relieve Urinary Incontinence Symptoms in Climacteric Women? A Feasibility Study

**DOI:** 10.1055/s-0041-1733979

**Published:** 2021-08-30

**Authors:** Anita Bellotto Leme Nagib, Valeria Regina Silva, Natalia Miguel Martinho, Andrea Marques, Cassio Riccetto, Simone Botelho

**Affiliations:** 1 Faculty of Medical Sciences, Statel University of Campinas, UNICAMP, Campinas, SP, Brazil; 2 University Center of Associated Colleges, São João da Boa Vista, SP, Brazil; 3 University José do Rosário Vellano, UNIFENAS, Alfenas, MG, Brazil; 4 Regional University Center of Espírito Santo do Pinhal, UNIPINHAL, Espírito Santo do Pinhal, SP, Brazil; 5 Physical Therapy Service, Center for Integral Attention to Women's Health – Women's Hospital Prof. Dr. José Aristodemo Pinotti, CAISM, State University of Campinas, UNICAMP, Campinas, SP, Brazil; 6 Postgraduate Program in Rehabilitation Sciences - Motor Science Institute - Federal University of Alfenas, UNIFAL-MG, Alfenas, MG, Brazil

**Keywords:** pelvic floor dysfunctions, urinary incontinence, pelvic floor muscle, gametherapy, climacteric, rehabilitation, disfunções do assoalho pélvico, incontinência urinária, músculos do assoalho pélvico, gameterapia, climatério, reabilitação

## Abstract

**Objective** To investigate the feasibility of pelvic floor muscle training (PFMT) through gametherapy for relieving urinary symptoms of climacteric women with stress or mixed urinary incontinence (UI).

**Methods** Randomized clinical trial, divided into two groups: Gametherapy (G_Game) and Control (G_Control). Both groups received recommendations about unsupervised PFMT, and G_Game also received supervised PFMT through gametherapy. After 5 consecutive weeks, the feasibility was investigated considering participant adherence, urinary symptoms (evaluated by the International Consultation on Incontinence Questionnaire-Urinary Incontinence Short Form [ICIQ-UI-SF] questionnaire), and pelvic floor function (PERFECT Scheme: power, endurance, repetition and fast). The Fisher exact, Kruskal-Wallis, Wilcoxon sign paired, and Mann-Whitney U tests were used by intention-to-treat analysis, using STATA 15.1 (StataCorp, College Station, TX, USA) software.

**Results** The present study included 20 women per group and observed a higher adherence in G_Game. In the intragroup analysis, a decrease in the ICIQ-UI-SF score was observed in both groups (14.0 to 10.0; 13.5 to 0), associated with increased endurance (2.5 to 3.5; 2.5 to 4.0) in G_Control and G_Game, respectively. Moreover, there was a concomitant increase in pelvic floor muscles (PFMs) power (2.0 to 3.0), repetition (3.0 to 5.0), and fast (10.0 to 10.0) in G_Game. In the intergroup analysis, a reduction of UI was observed ( *p* < 0.001; r = 0.8), as well an increase in PFM power ( *p* = 0.027, r = 0.2) and endurance ( *p* = 0.033; r = 0.3) in G_Game.

**Conclusion** The feasibility of supervised PFMT through gametherapy was identified by observing participant adherence, relief of urinary symptoms, and improvement in PFM function.

## Resumo

**Objetivo** Investigar a viabilidade do treinamento dos músculos do assoalho pélvico (TMAP) por meio de gameterapia no alívio de sintomas urinários em mulheres climatéricas com incontinência urinária (IU) de esforço ou mista.

**Métodos** Ensaio clínico randomizado, dividido em dois grupos: Gameterapia (G_Game) e Controle (G_Controle). Ambos os grupos receberam recomendações sobre TMAP não supervisionado, e G_Game também recebeu TMAP supervisionado por meio de gameterapia. Após 5 semanas consecutivas, a viabilidade foi investigada considerando a aderência das participantes, sintomas urinários (avaliados pelo questionário International Consultation on Incontinence Questionnaire-Urinary Incontinence Short Form [ICIQ-UI-SF]) e função do assoalho pélvico (esquema PERFECT: power, endurance, repetition, fast). Os testes exatos de Fisher, Kruskal-Wallis, sinal de Wilcoxon pareado e Mann-Whitney U foram usados pela análise de intenção de tratar, usando o software STATA 15.1 (StataCorp, College Station, TX, EUA).

**Resultados** O presente estudo incluiu 20 mulheres por grupo e observou uma maior adesão no G_Game. Na análise intragrupo, foi observada diminuição no escore do ICIQ-UI-SF em ambos os grupos (de 14,0 para 10,0; de 13,5 para 0), associada ao aumento da resistência (de 2,5 para 3,5; de 2,5 para 4,0) em G_Control e G_Game, respectivamente. Além disso, houve um aumento concomitante no power (de 2,0 para 3,0), repetition (de 3,0 para 5,0) e fast (de 10,0 para 10,0) dos músculos do assoalho pélvico (MAPs) no G_Game. Na análise intergrupos, foi observada redução da IU ( *p* < 0,001; r = 0,8), assim como do power ( *p* = 0,027; r = 0,2) e da endurance ( *p* = 0,033; r = 0,3) dos MAPs no G_Game.

**Conclusão** A viabilidade do TMAP supervisionado por meio de gameterapia foi identificada pela observação da aderência das participantes, pelo alívio dos sintomas urinários e pela melhora da função dos MAPs.

## Introduction

According to the International Continence Society (ICS), 1 urinary incontinence (UI) is defined as the involuntary loss of urine, and its importance lies rather in its impact on the quality of life in physical, psychological, and sexual aspects than in its related morbimortality. Stress urinary incontinence (SUI) is defined as the involuntary loss of urine on effort or physical exertion, while urgency urinary incontinence (UUI) is characterized by the involuntary loss of urine accompanied or immediately preceded by urgency, followed or not by other urinary symptoms such as frequency and nocturia. Mixed urinary incontinence (MUI) corresponds to the association of both types. Urinary symptoms like involuntary loss of urine are easily perceived and may be initially evaluated on clinical grounds only, using validated instruments that assess the impact of the condition. 2

The prevalence of UI reported among women ranges from 5 to 70%, and in postmenopausal women, > 40% of the population is affected. The prevalence of UI is strongly related to the age of women, so its appearance may increase with advancing age. 2 According to Dumoulin et al., 3 in the climacteric period, the modifications in the composition of pelvic floor muscles (PFMs) appear to affect their properties and their ability to function adequately, leading to UI.

Thus, the assessment of PFMs has been recommended by the ICS 1 as part of the clinical routine for investigating the PFMs function associated with the urogynecological signs and symptoms, and it is considered essential to assess the effects of the effect of the treatments performed. Digital palpation is one of the most practical and widely used methods due to its simplicity and low cost. 4 5

The conservative treatment of UI is recommended by the ICS as a first-line treatment, and it has been performed through pelvic floor muscle training (PFMT) with satisfactory levels of scientific evidence. 1 6 Recently, due to complications from midurethral mesh slings, less invasive alternatives have been considered for patients, who should be involved in the decision-making. 7 8

According to Dumoulin et al., 9 supervised PFMT is more effective than unsupervised PFMT. Supervised PFMT is considered the golden standard for the treatment of SUI and MUI, showing a high level of scientific evidence. 1 However, patient adherence to the training protocols represents the greatest threat to therapy success.

Gametherapy seems to be an interesting method to stimulate adherence to exercises due to the possibility of carrying out a training protocol that encourages and motivates the participants. In general, gametherapy is based on the combination of PFMT associated with a virtual game environment. Thus, the abdominopelvic musculature is stimulated while the patient interacts with the virtual platform, making the exercise pleasant, increasing the chances of adherence to the treatment. 10

Elliott et al. 11 showed the feasibility of PFMT for MUI through a virtual environment, using a computer with a dance game program (StepMania). Martinho et al., 10 Botelho et al. 12 and Silva et al. 13 developed a protocol capable of stimulating PFMs contractions during the execution of pelvic movements, induced by gametherapy.

The present study aimed at investigating the feasibility of supervised PFMT gametherapy for relieving UI symptoms and PFMs function in climacteric women with SUI or MUI when compared with an unsupervised protocol. We hypothesize that gametherapy may be an interesting, effective, and encouraging tool to be added to a conventional PFMT program, aiming at favoring adherence.

## Methods

### Study Design and Setting

The UroFisioterapia group of the Postgraduate Program in Surgical Sciences, Surgery Department, Division of Urology, Universidade Estadual de Campinas (UNICAMP, in the Portuguese acronym), Campinas, state of São Paulo, Brazil, performed a randomized clinical trial in the Physiotherapy Clinic of the Center for Integral Attention to Women's Health (CAISM, in the Portuguese acronym) of the UNICAMP.

The present study was approved by the Research Ethics Committee of the UNICAMP (Institutional Review Board approval CAAE: 41304914900005404; [U1111–1205–9058], Approval number 1.012.691) and was registered at ensaiosclinicos.gov.br following the CONSORT recommendations. 14 The participants of the study were instructed and informed about the procedures of the trial, and the ones who agreed formalized their acceptance by signing an informed consent form.

### Participants

Immediately after signing the informed consent form, the participants were submitted to the first assessment, in which the physical therapist researcher (Nagib A. B. L.), specialized in women's health, performed the investigation of clinical and demographic characteristics, as well as of urinary symptoms. Then, another physical therapist researcher (Martinho N. M.), also specialized in women's health, blind to the clinical and demographic characteristics of the participant, performed the physical PFMs examination.

The inclusion criteria were climacteric women (starting at 45 years old, a time when ovarian failure leads to decreased plasma steroid levels) 15 with dominant stress UI, assessed both by the International Consultation on Incontinence Questionnaire - Short Form (ICIQ UI-SF) 16 and by the International Consultation on Incontinence Questionnaire Overactive Bladder (ICIQ OAB), applied by the researcher responsible for the evaluation (Nagib A. B. L.). 17

The exclusion criteria were women with cognitive, neurological, and/or physical disorders that could hinder their participation in the assessment; current urinary tract infection (identified during the initial evaluation); a history of instrumental delivery, stress UI and/or surgical pelvic organ prolapse (POP), and oncology treatment and/or previous PFMT; inability to contract the PFMs (grade zero or 1, according to the Modified Oxford Grading Scale) 4 and/or POP > II, according to the POP quantification (POPq) 18 observed during the initial assessment; and inability to complete the initial assessment process.

### Interventions

The participants were randomized into two groups: Control Group (G_Control) and Gametherapy Group (G_Game). Both groups received recommendations on unsupervised PFMT. The G_Control was instructed to follow the recommendations of the unsupervised PFMT for 5 consecutive weeks and to return for the second evaluation. The G_Game received the same instructions as unsupervised PFMT and, additionally, participated in the supervised PFMT through gametherapy for 5 consecutive weeks, when they should return for the second assessment.

#### Control Group (G_Control)

The G_Control received only recommendations about unsupervised PFMT, performed by the main researcher, as follows: 1. PFM anatomy and function; 2. PFM control and coordination – performed during the assessment using digital palpation; 3. Delivery of a booklet about PFM control during daily activities. The instructions included the performance of PFM contraction exercises, at home (weeks 1 to 5), in different postures (laid down in the supine position with bent knees, seated, and squatting): A. Three sets with 10 maximum PFM contractions, ensuring 1 minute of rest between sets; B. Three sets of 10 moderate PFM contractions for up to 8 seconds. Then, relax gently. Keep 16 seconds of rest between sets; and C. Three sets of 10 quick contractions, followed by relaxation of the PFM, with 20 seconds of rest between sets. Moreover, the Knack Maneuver 19 20 was recommended, which consists in contracting the PFM before an activity involving physical effort (coughing, sneezing, carrying weight, and exercising) and when you are in a hurry to urinate (strong need to pee). The 5-week intervention protocol was established in an attempt to simulate the result at the end of 10 training sessions, which would correspond to most of the outpatient follow-up period in the clinical practice.

#### Gametherapy Group (G_Game)

**Fig. 1:**
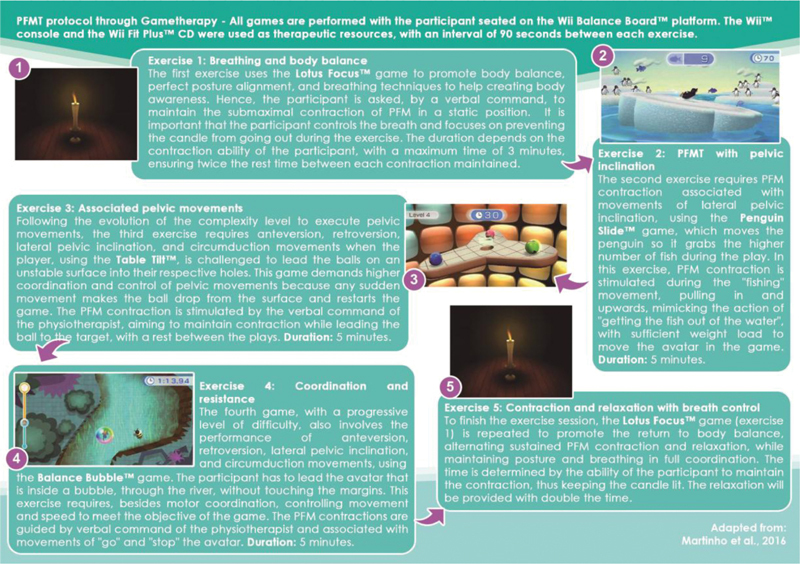
Supervised PFMT protocol through Gametherapy.

The G_Game received the same set of recommendations given to the G_Control and performed PFMT through gametherapy supervised by a physiotherapist (Nagib A. B. L.) twice a week for 30 minutes, for 5 consecutive weeks, resulting in 10 sessions. Later, a researcher specialist in physiotherapy (Martinho N. M.) the same who performed the first assessment, evaluated the group. The exercises were based on a specific gametherapy protocol developed by the research group, 10 11 12 13 using a Wii console with the Wii Fit Plus CD (games: Lotus Focus, Penguin Slide, Table Tilt, and Balance Bubble) and the Wii Balance Board platform. According to Martinho et al., 10 the game is controlled using pelvic exercises, with control and stabilization of the trunk. The volunteer remained seated on the Wii Balance Board platform, which was placed on a bench for the adequate maintenance and alignment at a 90° flexion of the knee joints and the hip. Then, the performance of retroversion, anteversion, and pelvic inclination was requested according to the avatar corresponding to the game played. For the present study, we added specific PFM contractions through verbal commands from the physiotherapist ( Fig. 1 ).

After 5 consecutive weeks, the participants in both groups performed the second evaluation, when urinary symptoms and the function of the PFMs were measured again. The frequency of exercises performed at home was monitored through the reports and notes of the participants.

## Outcomes

### Primary Outcome Measure

The relief of urinary symptoms after the treatment was the main clinical result. It was investigated by the validated questionnaire that investigates the severity of UI, using the Portuguese version of the ICIQ UI-SF, 16 which allows a quick investigation of the impact of UI on the quality of life and the measurement of urinary loss and interference with daily life, quantifying it from 0 to 21 (high scores mean more severe symptoms).

### Secondary Outcome Measure

The secondary outcome was measured based on the functional parameters of the PFMs. It was assessed with the “Power” of the PERFECT scheme, as proposed by Laycock et al., 4 and graduated according to the Modified Oxford Grading Scale, which allows the graduation of muscular strength, with a score ranging from zero to five, in which zero means absence of muscle contraction noticeable to the fingers of the examiner and five indicates strong contraction. 4

Another secondary result considered in the present study was feasibility, defined as the rate of adherence and completion of the protocol of the participant of both groups. To calculate adherence to the protocol, the participants had to perform the 10 PFMT sessions. Adherence to PFMT at home was calculated considering the frequency of exercises performed at home for 5 consecutive weeks, monitored through the reports and notes of the participants. The rate of completion of the PFMT protocol through gametherapy was calculated as the proportion of participants who completed the final assessment.

Additionally, the overactive bladder symptoms were investigated by the validated Portuguese version of the ICIQ–OAB, 17 which allows exploring the presence of frequency, nocturia, urgency and urgency UI, quantifying it from 0 to 16 (high scores mean more severe symptoms) in women with mixed UI.

### Randomization

The women included in the present study were randomly divided into two groups through a simple randomization process: control and experimental groups. The allocation of subjects was hidden by sequentially numbered, opaque, and sealed envelopes. After the assessment, the researcher opened the envelope attributed to each participant, following the treatment process. Every participant was aware of the possibility of being allocated to either one of the groups.

### Statistical Analysis

The analyses were conducted using the intention to treat (ITT) analysis methods; the variables presenting missing data were imputed with the last observation carried forward (LOCF) method.

The categorical variables were presented through absolute and relative frequencies, and they were compared with the Fisher exact test. All continuous variables were described and compared using medians and nonparametric methods, respectively, considering that they presented asymmetrical distribution. The medians of the control and intervention groups (comparison between groups) were compared with the Kruskal-Wallis rank test. The different moments of the study (pre- and postintervention) were compared with the paired Wilcoxon sign rank test. The intra- and intergroup effect sizes were also calculated with the paired Wilcoxon sign rank test and Mann-Whitney U statistics, respectively. The analyses were performed using Stata 15.1 software (StataCorp, College Station, TX, USA) at a 5% significance level ( *p* < 0.05). As suggested by Cohen, 21 the norms for interpreting the effect-size values were divided into “small” (0.1–0.3), “medium” (0.4–0.5), and “large” (> 0.5) effect, and we standardized the r to identify them.

## Results

**Fig. 2:**
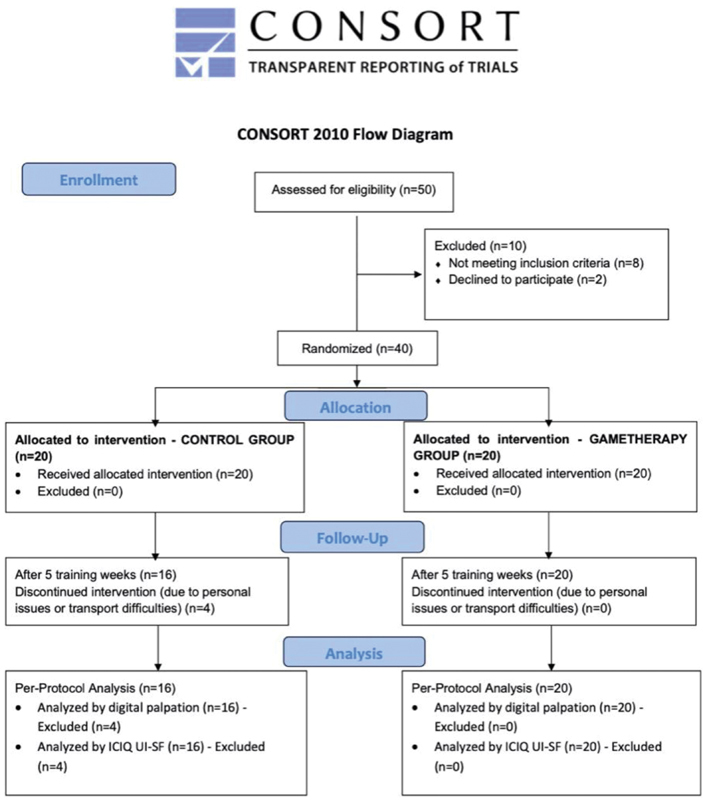
CONSORT flow diagram.

Initially, 50 women were recruited, evaluated, and distributed for treatment, according to Figure 2 . From these women, 40 were randomly divided for treatment between the groups. Adherence to the PFMT protocol was completed by 16 out of 20 (80%) participants in G_Control and by 20 out of 20 (100%) participants in G_Game, showing the highest adherence for G_Game. The completion rate of the PFMT protocol showed the same proportion as the adherence rate.

Table 1 presents the clinical and demographic characteristics of the participants. G_Control and G_Game were composed mostly of women of white color who were either married or cohabitating. Most of the women at G_Control had a primary school education. As for hormonal data, most women had menopause, but 16 (80%) did not undergo hormone replacement.

**Table 1:** Clinical and demographic characteristics of participants

	G_Control ( *n* = 20)		G_Game ( *n* = 20)		*p-value* ^a^
	N (%)	95%CI	N (%)	95%CI	
**DEMOGRAPHIC DATA**					
**Skin color**					
White	16 (80.0)	56.4– 92.5	18 (90.0)	66.5– 97.6	0.661
Other	4 (20.0)	7.5– 43.6	2 (10.0)	2.4– 33.5	
**Marital status**					
Single	1 (5.0)	0.7– 29.5	2 (10.0)	2.4– 33.5	1.000
Married/cohabitating	14 (70.0)	46.5– 86.2	13 (65.0)	41.8– 82.8	
Divorced/widower	5 (25.0)	10.5– 48.7	5 (25.0)	10.5– 48.7	
**Level of education**					
Illiterate	0 (0.0)	0.0– 0.0	0 (0.0)	0.0– 0.0	0.052
Elementary school	12 (60.0)	37.3– 79.1	6 (30.0)	13.7– 53.6	
High school	2 (10.0)	2.4– 33.4	10 (50.0)	28.7– 71.3	
Higher education	6 (30.0)	13.8– 53.5	4 (20.0)	7.4– 43.7	
**Physical activity**					
Does not practice	11 (55.0)	33.0– 75.2	9 (45.0)	24.7– 67.1	0.752
Up to twice a week	9 (45.0)	24.7– 67.1	11 (55.0)	32.9– 75.3	
**HORMONAL DATA**					
**Menopause**					
No	8 (40.0)	20.9– 62.7	4 (20.0)	7.4– 43.7	0.301
Yes	12 (60.0)	37.3– 79.1	16 (80.0)	56.3– 92.6	
**Hormonal replacement**					
No	16 (80.0)	56.4– 92.5	16 (80.0)	56.3– 92.6	1.000
Yes	4 (20.0)	7.5– 43.6	4 (20.0)	7.4– 43.7	
**OBSTETRIC DATA**					
**Pregnancies**					
0	1 (5.0)	0.7– 29.5	3 (15.0)	4.7– 38.6	0.094
1	2 (10.0)	2.4– 33.4	3 (15.0)	4.7– 38.6	
2	3 (15.0)	4.7– 38.6	8 (40.0)	20.9– 62.7	
3	14 (70.0)	46.5– 86.2	6 (30.0)	13.7– 53.6	
**Number of vaginal deliveries**					
0	11 (55.0)	33.0– 75.2	8 (40.0)	20.9– 62.7	0.546
1	1 (5.0)	0.7– 29.5	3 (15.0)	4.7– 38.6	
2–3	8 (40.0)	20.9– 62.7	9 (45.0)	24.7– 67.1	
**Number of cesarean deliveries**					
0	9 (45.0)	24.7– 67.1	10 (50.0)	28.7– 71.3	0.054
1	2 (10.0)	2.4– 33.4	7 (35.0)	17.2– 58.2	
2–3	9 (45.0)	24.7– 67.1	3 (15.0)	4.7– 38.6	
**PERSONAL DATA**	**Median**	**IQR**	**Median**	**IQR**	***p-value*** ^b^
Age (years old)	49.5	41.0– 61.0	57.0	51.5– 61.0	0.116
BMI (kg/m2)	25.4	21.7– 30.5	24.6	22.0– 29.2	0.818

Abbreviations: BMI, body mass index; CI, confidence interval; IQR, interquartile range.

The table presents demographic and clinical data expressed in percentage (%) followed by the confidence interval, using the ^a^ Fisher's exact test and considering a 95% confidence interval. It also presents personal data expressed in median and interquartile range, using the ^b^ Kruskal-Wallis rank test and considering *p* < 0.05.

Table 2 shows the urinary symptoms assessed by the ICIQ UI-SF and ICIQ–OAB questionnaires, comparing both times (intragroup analysis) and groups (intergroup analysis). All participants observed relief of urinary symptoms.

**Table 2:** Comparison of urinary symptoms in both groups, pre- and postintervention, using the ICIQ UI-SF and ICIQ-OAB questionnaires, by intention to treat analysis

	Intragroup analysis								Intergroup estimates	
	G_Control ( *n* = 20)				G_Game ( *n* = 20)					
	Median	IQR	*p-value* ^a^	Effect size ^c^	Median	IQR	*p-value* ^a^	Effect size ^c^	*p-value* ^b^	Effect size ^d^
**ICIQ UI-SF**										
Preintervention	14.0	12.0–15.0	**< 0.001**	0.317	13.5	12.0–17.5	**< 0.001**	0.393	0.560	0.446
Postintervention	10.0	6.0–12.5			0.0	0.0–4.5			**< 0.001**	0.863
**ICIQ-OAB**										
Preintervention	4.5	2.0–8.0	0.058	0.194	3.0	1.0–5.5	**0.002**	0.290	0.317	0.593
Postintervention	3.0	1.5–5.5			2.0	1.0–3.0			0.074	0.665

Abbreviations: ICIQ-OAB, International Consultation on Incontinence Questionnaire Urinary Incontinence - Overactive Bladder; ICIQ UI-SF, International Consultation on Incontinence Questionnaire Urinary Incontinence - Short Form; IQR, interquartile range.

The table presents comparisons between pre- and postintervention periods, comparing the times ( ^a^ Wilcoxon rank test) and groups ( ^b^ Kruskal-Wallis rank test). The data are presented with medians and interquartile range, according to intention to treat analysis. Effect sizes were calculated both for intra- ( ^c^ Paired signed-rank Wilcoxon test) and intergroup comparisons ( ^d^ Mann-Whitney U statistic).

Table 3 presents PFMs strength measured by digital palpation, comparing both times (intragroup analysis) and groups (intergroup analysis). There was an increase in PFMs strength in patients who underwent gametherapy exercises.

**Table 3:** Investigation of pelvic floor muscles function in both pre- and postintervention groups, through digital palpation, transperineal ultrasound (4D TLUS), and sEMG, by intention to treat analysis

	Intragroup analysis								Intergroup estimates	
	G_Control ( *n* = 20)				G_Game ( *n* = 20)					
	Median	IQR	*p-value* ^a^	Effect size ^c^	Median	IQR	*p-value* ^a^	Effect size ^c^	*p-value* ^b^	Effect size ^d^
**Power**										
Preintervention	2.0	2.0–3.0	0.250	0.173	2.0	2.0–2.0	**< 0.001**	0.407	0.256	0.605
Postintervention	2.5	2.0–3.0			3.0	3.0–3.0			**0.027**	0.295
**Endurance**										
Preintervention	2.5	2.0–4.0	**0.016**	0.256	2.5	2.0–3.0	**< 0.001**	0.383	0.598	0.549
Postintervention	3.5	2.5–4.0			4.0	3.5–6.0			**0.033**	0.302
**Repetition**										
Preintervention	3.5	2.5–5.0	0.114	0.163	3.0	3.0–4.0	**< 0.001**	0.376	0.579	0.551
Postintervention	4.0	3.0–6.0			5.0	4.0–6.5			0.055	0.323
**Fast**										
Preintervention	10.0	7.0–10.0	0.922	0.031	10.0	5.0–10.0	**0.008**	0.280	0.607	0.547
Postintervention	10.0	7.0–10.0			10.0	10.0–10.0			0.250	0.394

Abbreviation: IQR, interquartile range.

The table presents comparisons between pre- and postintervention periods, comparing the times ( ^a^ Wilcoxon rank test) and groups ( ^b^ Kruskal-Wallis rank test). The data are presented with medians and interquartile range, according to intention to treat analysis. Effect sizes were calculated both for intra- ( ^c^ Paired signed-rank Wilcoxon test) and intergroup comparisons ( ^d^ Mann-Whitney U statistic).

## Discussion

According to the results obtained in the present study, all participants observed relief of urinary symptoms and increased PFMs endurance. However, the increase in PFMs power, repetition and fast was verified only in supervised PFMT through gametherapy. As described by Braekken et al., 22 the improvement in PFMs contractility after PFMT increases muscle volume, contributing to their support, resistance, and coordination, and improves PFMs functionality, which is an important aspect to show the effectiveness of PFMT, as indicated by Bø et al. 23 Based on this premise, we assume that the gametherapy protocol provided not only the relief of incontinence symptoms but also a significant increase in PFMs function.

However, we believe that a PFMT program performed routinely, especially the one “learned” after a “complete” pelvic floor evaluation, could promote an increase in PFMs activity, contributing to greater pelvic floor support and closure of the urethral sphincter, also improving the control of urinary symptoms, especially when combined with the precontraction test (The Knack). 20

The execution of PFMs precontraction during everyday activities that involve increasing intraabdominal pressure may have a fundamental role in preventing future dysfunctions, which leads to an improvement in the quality of life. However, despite providing knowledge about these muscles and the possibility of their recruitment during functional activities, this exercise does not guarantee a significant effect on the maximum contraction capacity of the PFMs. 24 Henderson et al. 25 evaluated 779 women through the Brinks Scale and showed that most women with or without mild PFMs disorders are capable of correctly contracting those muscles after simple verbal orientation. We believe that, in the present study, both the reduction of urinary symptoms and the increase in PFMs strength were due to the recommendations received during the physiotherapy assessment and to the booklet of domestic recommendations that both groups received.

Hung et al. 26 affirms that learning from training can modify muscular recruitment, with consequent improvement in the coordination between PFMs and abdominal muscles, considering that the pelvic floor works coordinately with the stabilizing abdominal muscles, promoting closure of the urethral sphincter after receiving effort commands from the upper part of the body. 27

Gametherapy has been used as a rehabilitation technique in multiple healthcare fields. 28 Studies by Martinho et al., 10 Elliott et al., 11 Botelho et al. 12 and Silva et al. 13 have shown that this tool can be complementary to PFMT, which could stimulate the adherence of patients due to the sensorial feedback, easy handling, and low cost. Among the studies performed, positive results were observed in both asymptomatic young women and incontinent women.

Elliott et al. 11 investigated the viability of using PFMs strengthening exercises associated with virtual reality in elderly women with UI. The authors concluded that the association of the exercises was effective to improve symptoms and quality of life.

The gametherapy protocol used in the present study corroborates the study by Silva et al. 13 performed with continent nulliparous young women. The authors verified an increase in muscle strength identified by vaginal palpation and improvement in the coactivation of PFMs in response to abdominal contraction. According to the authors, one of the challenges of the preventive practice in this area refers to the introduction of proposals that emphasize the importance of the awareness of abdominopelvic muscles as a type of prevention against pelvic floor overload during daily activities.

Additionally, Martinho et al. 10 observed an increased PFMs strength and ability to maintain contraction in the reduction of urinary symptoms, which reflected in the improvement of PFMs functionality and quality of life in postmenopausal women. The authors observed good acceptance, easy applicability, and treatment continuity.

The current protocol was based on the one performed by Martinho et al. 10 and Silva et al., 13 which consisted of the addition of PFM contraction at the time of exercise practice using virtual games with the training focused on PFM contraction, attending to the specificity principle as recommended by the Physical Activity Guidelines Advisory Committee. 29 Still according to the Committee, 29 as the participant familiarizes with the game (training adaptation), gametherapy provides higher interactivity through the increase in the level of execution, attending to the overload principle. This principle is defined as the physical stress applied to the body when physical activity is more intensive than usual, reflecting on the adaptation of body structures and functions as a response to stimuli.

The maintenance of the effect of the treatment is secondary to the continuity of the training proposed, which requires attention to the frequency, intensity, and duration of exercises. These parameters are easily programmed in gametherapy, with precision in reproducing the techniques proposed.

Adherence to training is still the greatest challenge to overcome, considering that the dropout rate could harm the results obtained during the treatment. Porta Roda et al. 30 identified that low adhesion may occur even in an efficient exercise program. Considering that PFMT requires adherence, the supervision by a trained professional tends to increase motivation and, consequently, the adherence to the treatment program, besides providing better control in the execution of the techniques proposed.

The association of supervision by a professional physiotherapist with the exercise program may have contributed positively to the findings of our study. 31 Moreover, higher assiduity was observed in the experimental group, which suggests that gametherapy may be more attractive and increase the adherence of women.

Another relevant aspect of adherence refers to motivation, which is an essential condition for the assiduity of participants in the exercise program. Araujo et al. 32 used a digital app as a guide for PFMT and compared it with the control group, which received only written PFMT instructions. The authors observed that app use increased PFMT adherence in women with UI.

These findings suggest that training programs using innovative instruments combined with motivation and adequate understanding of the exercises may stimulate PFMs and increase the chance of adherence to treatment. 13 33

The present study suggests that exercises performed with gametherapy could increase PFMs strength and reduce urinary symptoms in women with a prevalence of SUI. However, new studies with a higher number of participants could establish a better understanding of these benefits.

As a limiting factor, we consider the small number of subjects in our sample. Furthermore, the individualized patient-therapist contact in the experimental group does not exclude the possibility of a greater endeavor in performing the exercises, as shown in previous supervised studies. The reduced period of training could have affected the findings, considering that some authors 31 recommend the performance of PFMT for a minimum of 6 weeks for adaptation. The performance of further studies is recommended to elucidate the effects of different kinds of treatment on the anatomic and functional conditions of this population. Therefore, supervised gametherapy is probably better than a nonsupervised program, but future studies should assess whether this novel approach is advantageous when compared with traditional individual supervised sessions of PFMT. According to Nagib et al., 34 the use of apps could be an important tool to assist future studies in controlling the adherence of the participants to the training protocol. Although our study did not detect any statistical difference between the participants regarding hormone replacement, we emphasize that hormone replacement should be carefully evaluated in future studies to avoid the possibility of bias.

In addition, we also consider important to monitor attendance at home, but it is important to emphasize how difficult this is. With the advent of technology, it is possible that, in the future, some means of monitoring adherence to home exercises may be feasible, promoting a greater control of the results.

We observed that all women presented relief in urinary symptoms and increased PFMs endurance. However, those who performed the supervised training program using gametherapy also improved other domains of the PERFECT scheme (power, repetition and fast), which shows the feasibility to add gametherapy as a tool for PFMT.

## Conclusion

The feasibility of supervised PFMT through gametherapy was identified, and adherence of the participants, relief of urinary symptoms, and improvement in PFMs function were observed.
